# Supplementary material for: Are carbon emissions trading and green financial instruments synergistic? -Comprehensive quantitative research based on content analysis
Source: PLoS One. 2024 Mar 7;19(3):e0298601. doi: 10.1371/journal.pone.0298601 (PMC10919601; doi:10.1371/journal.pone.0298601)
Supplement: S1 Data — (ZIP) [file pone.0298601.s001.zip › Data/地级市Z_F工作报告.zip!/╣π╢1⁄2/╓Θ║ú╩╨/└┤╫╘╣1⁄2╓┌║┼í╛┬φ┐╦╩2╛▌═°í┐_2016.docx]

各位代表：

　　我代表市人民政府，向大会报告政府工作，请予审议，并请市政协委员和其他列席人员提出意见。

　　一、“十二五”时期及2015年工作回顾

　　“十二五”时期，是珠海发展史上开创性的五年。在市委的正确领导下，在市人大、市政协的监督支持下，我们积极应对国际金融危机的持续影响，主动适应经济发展新常态，深入实施《珠江三角洲地区改革发展规划纲要》，统筹推进交通、产业、城市、社会和民生建设，较好地完成了“十二五”确定的目标任务，为“十三五”发展奠定了良好基础。

　　五年来，我们积极应对经济下行压力，找准稳增长和调结构的平衡点，制定实施了培育大型骨干企业、促进外经贸转型发展、壮大民营经济和总部经济等一系列政策措施，经济发展提速提质。地区生产总值从“十一五”末的1211亿元跃升到2038亿元，人均地区生产总值约合2万美元，迈入世界高收入经济体行列。固定资产投资、一般公共预算收入均比“十一五”末翻了一番，年均增速居珠三角前列。

　　五年来，我们牢记特区使命，将改革开放作为新常态下加快发展的最大动力，全力破解体制机制障碍，加快构建开放型经济新体制，打造市场化国际化法治化营商环境，经济社会发展活力明显增强。横琴新区开发建设实现五年巨变，步入自贸区时代，以珠港澳合作为重点的对外开放实现新突破。商事制度改革、农村综合改革、信用体系建设、社会治理创新等走在全国前列。

　　五年来，我们顺应全球产业发展新态势，抢占产业制高点，以创新驱动发展为核心战略，全力建设“三高一特”现代产业体系，产业水平大幅提升。高新技术产品产值占规模以上工业总产值比重提升到55%，现代服务业增加值占服务业比重提升到57.6%，均居全省前列。

　　五年来，我们全力扭转交通末梢地位，交通建设进入投入最多、建设规模最大的时期。港珠澳大桥加快建设，珠海将成为重要的国际门户城市。广珠铁路、广珠城轨通车，结束了珠海无铁路的历史。珠海港被确定为国家综合运输体系的重要枢纽，迈入亿吨大港行列。机场旅客年吞吐量达到470万人次，是“十一五”末的2.6倍。横琴口岸实现24小时通关，拱北口岸和珠澳跨境工业区专用口岸实施延关。

　　五年来，我们有序铺开国际宜居城市建设。成功创建全国文明城市、国家生态市和国家生态园林城市，连续两年被评为中国宜居城市第一名。西部生态新区升级为省级发展平台。香洲社区体育公园和横琴新区综合管廊荣获2015年中国人居环境范例奖。圆满完成“十二五”节能减排任务，环境空气质量在全国74个重点城市中保持前十水平。

　　五年来，我们坚持把提升市民幸福指数作为最大的价值追求，全市人民得到更多实惠。市、区两级财政累计投入792亿元用于基本民生支出，教育文化、社会保障保持全省领先水平。幸福村居建设实现三年大变，农村常住居民人均可支配收入比“十一五”末增长80.6%，城乡居民的生活质量明显改善。

　　总之，通过“十二五”的努力，全市的综合实力和发展水平迈上了新的台阶，珠海站在了新的历史起点上。

　　各位代表，2015年初召开的市八届人大五次会议，审议批准了市政府提出的8个方面217项具体工作。根据发展情况的变化，市政府又新安排了28项具体工作。经过一年的努力，上述245项工作中，240项已经完成，1项作了调整，4项进度相对滞后，正在抓紧落实，将在2016年内全部完成。具体情况在附件一中有详细报告。初步测算，2015年全市实现地区生产总值2038亿元，比上一年增长10%，增速居全省第一；一般公共预算收入269.9亿元，增长17.2%；规模以上工业增加值980亿元，增长9.7%；全体居民人均可支配收入达到36093元，增长8.6%；城镇登记失业率处于2.26%的较低水平；居民消费价格涨幅为1.7%；十件民生实事基本完成。成功举办了首届“装洽会”、WTA超级精英赛、第二届世界广府人恳亲大会、第二届中国国际马戏节等大型活动。全市呈现经济质效提升、民生持续改善、社会和谐稳定的良好态势，实现了“十二五”的圆满收官。

　　一年来，我们重点做了六项工作：

　　（一）努力保持经济持续向好势头

　　扩大有效投资和消费，稳定外贸增长。完成固定资产投资1305亿元，增长15%，其中112个重点建设项目完成投资423.4亿元。实际吸收外商直接投资21.8亿美元，增长12.8%。培育新型消费模式和消费热点，社会消费品零售总额913.6亿元，增长12%。

　　外贸进口由于原油等大宗商品价格大幅下降而下滑了26.8%，出口在极其困难的环境下增长0.6%。

　　深入开展扶企强企行动。颁布促进民营经济发展条例，出台支持小微企业上规模政策，新增规模以上企业38家。落实各项税收优惠政策，清理进出口环节收费项目109项，公布政府定价涉企经营服务收费目录清单和政府性基金目录清单，为企业减免税费84亿元。

　　加快发展以先进装备制造业为重点的高端产业。促成三一海洋重工等18个重点项目投产，新兴重工应急救援装备产业园等13个重大项目开工，福陆海工等28个项目签约落户，完成装备制造业投资128亿元。启动工业企业转型升级和技术改造三年行动计划，完成技改投资89.6亿元，增长超过50%。全市装备制造业增加值达到376亿元，增长15.5%。金融业预计实现增加值135亿元，增长14%，占GDP的6.6%，持牌法人金融企业新增10家，达到58家。

　　发展面向未来的新产业、新业态。格力国际智能制造基地加快建设，高新区机器人产业园动工，全球机器人领军企业ABB落户高新区。跨境电商通关监管场所和综合服务平台建成启用，阿里巴巴“全球货源”珠海站、聚美优品华南总部等项目落户，全市电子商务交易额超过900亿元。长隆第二主题公园开工，嘉华集团体育休闲度假中心、华彬珠海健康中心、航空大世界等高端消费类项目落户。全年接待过夜游客1330多万人次，旅游总收入增长4%。

　　（二）全面谋划实施创新驱动发展战略

　　把创新驱动发展确立为核心战略，系统谋划创新驱动总体布局和路线图，制定了市、区两级三年行动计划，明确了“8个倍增”目标。学习借鉴深圳经验，出台促进科技创新18条核心政策，形成“1+5”创新驱动政策体系。全市新增高新技术企业51家，总量达到397家，入选省高企培育库企业196家。新增上市企业1家、“新三板”挂牌企业37家。

　　加强公共创新平台建设。新增孵化器建筑面积31万平方米，新增在孵企业200多家。一批众创空间快速兴起，吸引154个创业团队进驻。清华大学珠海创新中心、华南理工大学珠海现代产业研究院等6家新型研发机构挂牌成立，国家船舶及海洋工程装备材料质量监督检验中心投入使用。与中山大学等5所高校及科研院所开展战略合作。新增51家工程中心、企业技术中心，其中省级26家。组织实施省重大科技项目25个、科技型中小企业创新项目51个，全社会研发投入达到55亿元，占GDP比重达2.7%。高新区在全国排位上升10位。

　　完善金融、人才等服务支撑体系。组建珠海科技创业投资公司，引进股权（创业）投资企业799家，“新三板”华南服务基地落户横琴，科技金融广场成功对接项目349个。全面实施蓝色珠海高层次人才计划，引进国家“千人计划”专家13人、诺贝尔奖得主2人和创新创业团队5个，4人入选省领军人才，居全省地级市首位。设立横琴国际知识产权交易中心、知识产权巡回法庭和知识产权检察工作站，在横琴自贸试验区初步建立起知识产权交易和保护机制。

　　（三）启动横琴自贸试验区建设

　　对接高标准国际投资贸易规则。在外商投资管理、商事制度等领域实行118项创新举措，出台全国首个促进自贸试验区建设的政府规章，制定政府部门权力清单、市场主体违法行为提示清单、横琴与港澳差异化市场轻微违法经营行为免罚清单。实行小规模纳税人简并征期，推出“三个零”政府服务。推行立案登记制，建立类似案例辩论制度和第三方法官评鉴制度。珠海国际仲裁院、珠港澳商事争议联合调解中心正式运作。政府智能化监管服务模式入选全国自贸试验区“八大最佳实践案例”，一批改革创新措施在全省推广。

　　落实对港澳服务业扩大开放措施。编制港澳投资准入负面短清单，粤港澳服务贸易自由化省级示范基地挂牌，1238家港澳投资企业落户横琴。开展“一机一台”通关模式改革，实行澳门小商品通关简化归类。中国-拉美企业家理事会联络办公室落户，113个项目入驻横琴澳门青年创业谷。

　　推进金融开放创新。跨境人民币贷款政策落地，企业申请备案的跨境人民币贷款超过53亿元。新增金融类企业1300家，各类要素交易平台发展到11家，全省第二家金融租赁公司华通金融租赁有限公司开业运营。国家食品安全（横琴）创新中心挂牌，国家“互联网+”创新创业基地落户，国家中医药现代化科技产业创新联盟、中国中医科学院中医药国际创新中心在横琴成立。

　　（四）以深化改革激发发展活力

　　推进简政放权。向区一级下放182项行政管理事权，赋予各区更多发展自主权。取消和调整行政审批事项45项，清理非行政许可审批事项244项，不再保留非行政审批类别。完成“一照一码”商事登记制度改革，日均新登记注册商事主体超过200户，每千人商事主体数量增加24户，达到144户。

　　简化投资审批流程。实行“一个窗口”受理审批投资立项制度，试行建设项目快速落地机制，推行网上备案立项制度，建设工程可行性研究报告审批时限压缩至12个工作日以内。实行招标核准负面清单制度，减少90%的招标核准事项。制定政府与社会资本合作三年行动方案，确定了一批PPP模式的建设项目。

　　切实推动重点改革。启动市、区两级财政零基预算改革。实施不动产统一登记。完成农村集体建设用地和留用地使用权确权登记试点工作。深化基层自治，公布社区行政事务禁入目录。推出公安改革创先“10+1”惠民项目。健全多元化纠纷解决机制，建立社会评议委员会制度，村居警官、法律顾问覆盖全市村居。建立安全生产联合执法机制，各类生产安全事故宗数同比下降4.24%。

　　（五）深入推进国际宜居城市建设

　　推进交通工程建设。港珠澳大桥珠海口岸主体工程封顶。横琴二桥建成通车，月环支线延长线建设进入收尾阶段。香海大桥、洪鹤大桥先行段开工建设。洪湾枢纽互通二期、白石桥全面施工。珠海至北京、桂林跨省列车开通。珠海口岸查验机制创新试点获批。洪湾港二期码头项目建成运营，九洲港、香洲港货运码头完成历史性搬迁。高栏港15万吨级主航道、5万吨级石化码头和三一重工配套码头建成运营，川贵粤-南亚物流大通道项目启动。珠海机场对外籍公务机临时开放口岸获批，通用机场项目获批并投入建设。东西部快速公交系统着手建设。建成东部城区慢行系统89公里主廊道。新增314个公共自行车服务站点。建成5座人行立体过街设施。

　　加快东西城区核心区建设。改造提升核心城区，基本完成情侣路海滨泳场、香炉湾和凤凰湾沙滩修复工作，城市之心一期动工建设，新一轮旧村改造全面启动。签署珠中两市合作治理前山河协议，拱北污水处理厂扩建工程建成运营，前山污水处理厂一期工程顺利封顶，流域第一批28个污水管网工程和23条旧村截污工程全面动工。铺开西部生态新城建设，完成8286亩土地清理工作，全面展开起步区市政基础设施建设，建成9条市政道路，启动海绵城市示范区和地下综合管廊建设。

　　扎实建设幸福村居。农村生活垃圾和污水处理设施基本覆盖所有行政村，146个村居被评为省级卫生村。乡村旅游接待游客超过600万人次，十里莲江乡村旅游风情带入选全国十大乡村游精品线路，南门村、莲江村获评中国乡村旅游模范村。农业部去农庄网华南运营中心、阿里巴巴农村淘宝项目落户斗门。居家养老服务覆盖90%以上的村居，基本养老保险制度覆盖全市农业户籍人口。

　　加强环境保护和生态修复。强化大气污染防治，空气质量达标率达到90%，提高1.6个百分点，PM2.5、PM10年均浓度降至31和51微克/立方米，降幅分别为8.8%和3.8%。划定超过1000平方公里生态控制区，新建成1.19万亩碳汇林、73.3公里生态景观林带，新增绿道80公里，人均公园绿地面积达到19.5平方米。入选中欧低碳生态综合试点城市和国家水生态文明建设试点城市。

　　（六）提升基本公共服务水平

　　深化教育综合改革。市直幼儿园和初中全部移交区级政府管理。公办幼儿园学位全部公开摇号派位，高中入学全面取消择校生。组建职业教育集团，出台促进民办教育规范特色发展实施办法，开展校长职级制评审。建成第二批10所镇中心幼儿园、斗门特殊教育学校等教育设施。平安校园达标率达到92.6%。成功创建省推进教育现代化先进市。

　　推进公立医院、基层医疗卫生机构和办医体制改革。公立医院统一实行药品和医用耗材零差率政策。实行社区卫生服务标准化，推广家庭医生制度。区域医疗一卡通建成。“单独两孩”政策顺利实施。计划生育证明不再作为出生入户的前置条件。市人民医院北区投入使用，省中医院珠海医院、市第二中医院（侨立中医院）住院楼主体完工。启动食品安全城市创建试点工作。实施家禽集中屠宰、冷链配送、生鲜上市，83家农贸市场完成改造升级。通过国家卫生城市复审。

　　提升公共文化服务水平。建设和改造一批基层文化站、村居文化中心、数字农家书屋、社区体育公园和镇级全民健身广场。成功举办莫扎特国际青少年音乐比赛等一批国际性文体盛事。珠海大剧院、博物馆和规划展览馆主体工程基本完成，成为珠海的文化新地标。

　　提升基本民生保障水平。城乡居民医疗保险财政补贴标准提高到每人每年400元，困难群众住院自付医疗费用救助比例达到80%以上，重特大疾病最高救助金额达到每人每年20万元。小学、初中公用经费补助标准分别提高到每人每年1150元和1950元。公共卫生服务经费财政补助标准提高到每人每年45元。低保标准提高到每人每月580元。集中供养、分散供养孤儿基本生活标准分别提高到每人每月1300元和870元。免费乘坐公交覆盖非本市户籍老人。城镇新增就业4.8万人。基本建成3986套保障性住房，新开工4439套。圆满完成市内外扶贫开发工作任务。对口帮扶阳江工作在全省考核中排名第一，珠海（阳江）产业园和珠海（阳江万象）产业园均获省考评优秀。

　　一年来，政府系统大力改革影响效率的工作机制，深入开展“三严三实”专题教育，领导干部更多地深入一线解决问题、推动工作，政府的行政效率和执行力明显提升。市政府更加注重加强和改进政府立法，出台《政府规章立法程序实施办法》《政府立法公众参与办法》，建立政府立法人才库，提请市人大审议地方性法规草案10件，制定政府规章6件。更加注重完善依法决策机制，制定《重大行政决策程序规定》，健全重大行政决策听证指导和监督制度，举行了10场重大行政决策听证会。更加注重党风廉政建设和巡视整改，出台《预防腐败条例实施细则》及30多项配套制度，政府投资工程廉情预警评估电子化信息平台、财政专项资金申报和管理平台投入使用，实现政府投资项目全程监督。更加自觉地接受人大和政协监督，完善人大预算实时在线监督系统，修订人大议案、代表建议和政协提案办理办法，完成577件建议和提案的办理答复工作，主动约见部分市人大代表听取意见，四小联围海堤达标工程议案顺利结案。更加注重利用公共传媒广纳民意，首次向社会公开征集民生实事建议。

　　各位代表，每一届政府都不可避免地遗留当时历史条件下难以解决的问题。一届负责任的政府，必须积极面对和解决这些问题。过去一年，我们争取了省的政策支持，通过自愿补缴的方式，将被征地农民等群体纳入职工养老保险，政策惠及4.6万人。我们制定了农村集体留用地落地工作方案，首批已落实2300亩。我们全面梳理、打通断头路，宝翠桥工程、人民西路与坦洲环洲东南路衔接工程完工，金唐西路复工，兰埔路改造项目进场施工。对烂尾楼和土地开发的历史遗留问题，我们也逐一研究制定了解决方案。

　　各位代表，需要特别报告的是，梅华路有轨电车1号线没有按预定的时间投入运营，主要是对项目技术的成熟度论证不充分。对此，市政府承担主要责任。我们将以此为鉴，进一步提高重大公共决策的民主化和科学化水平。目前，经过组织技术攻关，项目的技术问题已基本解决，并开始试运行，3月初将对公众开放试乘。我们将完善后续工作，尽早实现正常运营。

　　各位代表！珠海发展取得的一切成就，都归功于全市人民的共同努力。在这里，我代表市政府，向关心城市发展、为城市进步辛勤工作的市民朋友们，表示崇高的敬意！向给予政府工作大力支持的人大代表和政协委员，向各民主党派、工商联、无党派人士和人民团体，向中央和省驻珠单位，向驻珠部队和武警官兵，向关心支持珠海发展的港澳台同胞、海外侨胞和国际友人，一并表示衷心的感谢！

　　回顾过去的一年，我们也清醒地看到，政府工作仍然存在值得警醒和需要解决的突出问题。一是营商环境仍然存在不少瓶颈环节，行政审批流程复杂、效率不高的问题突出，办事难依然是企业和群众反映强烈的问题。二是体制机制改革还不到位，市场的活力没有充分释放，基层的积极性没有充分发挥，政府风险防控还存在薄弱环节。三是部分领导干部不愿担责、不敢担当的问题突出，一些重点工作进展滞后，社会关注度高的问题没有及时得到解决。四是对群众反映强烈的民生问题，特别是中心城区交通拥堵、东西部之间交通建设滞后、规范化普惠性幼儿园严重不足等突出问题，政府还没有更加有效的解决措施。对此，我们将高度重视，在新一年工作中制定切实措施逐项解决。

　　二、“十三五”时期奋斗目标和主要任务

　　“十三五”时期是全面建成小康社会、实现第一个百年奋斗目标的决胜阶段，也是珠海加快建设生态文明新特区、科学发展示范市的关键五年。虽然国际金融危机的深层次影响还在持续，外部经济下行压力加大，我们自身也面临着创新驱动基础薄弱、东西部发展不平衡、交通基础设施不完善等困难和挑战。但总体而言，我们正处于加快发展的黄金机遇期，特别是横琴自贸试验区和珠三角国家自主创新示范区的设立，港珠澳大桥和深中通道的建设，珠江西岸先进装备制造产业带战略的实施，都为珠海发展带来了叠加利好。经过“十二五”的努力，珠海新的增长动力正在孕育，持续向好的发展态势已经形成，外界也普遍看好珠海的发展。只要我们抓住机遇，强化责任担当，狠抓工作落实，一定能实现更有质量、更高水平的发展。

　　根据《中共珠海市委关于制定国民经济和社会发展第十三个五年规划的建议》，市政府编制了《珠海市国民经济和社会发展第十三个五年规划纲要（草案）》，并对《纲要》的起草情况作了说明，提交本次会议审议。

　　“十三五”时期全市发展的主要预期目标是：率先在2017年全面建成小康社会，率先向基本实现社会主义现代化阔步迈进。“十三五”期间提前实现地区生产总值和全体居民人均可支配收入比2010年翻一番。地区生产总值年均增长9%，到2020年超过3000亿元，人均达3万美元。

　　地区生产总值年均增长确定9%的预期目标，主要是考虑新常态下将更加注重发展质量和效益，更加注重结构优化和动力转换，必须适当提高换挡降速的容忍度。同时，我们也通过多种模型进行了测算。结果表明，确定这个增长目标是合理的，也是必要的。

　　对照国家统计局全面建成小康社会统计监测指标体系，珠海市小康指数已达到95%左右，已总体上接近小康社会水平。综合考虑发展基础和特区责任，我们认为，2017年率先全面建成小康社会，比全省提前一年，是合理可行的。只要我们在今后两年全力补齐居民人均可支配收入、家庭人均住房面积达标率、居民消费支出占GDP比重等短板指标，就能够实现这一目标。

　　完成“十三五”各项目标任务，必须崇尚创新、注重协调、倡导绿色、厚植开放、推进共享。需要重点在七个方面下功夫：

　　一是建设国际化创新型城市。全面实施创新驱动发展的核心战略，实施三年行动计划，完善创新创业政策法规，构建国际化创新体系，充分发挥高新区和横琴自贸试验区在创新中的核心作用。推进科技企业孵化器倍增计划，重点培育一批具有国际竞争力的创新型企业，全市高新技术企业超过800家。推进新型研发机构和公共平台建设，深化与中山大学、吉林大学和省科学院等产学研合作，新增一批国家级创新平台。培育发展创新型金融，推进横琴新区“三链融合”发展示范区和高新区科技金融试点建设。实施高层次人才倍增计划，推进横琴国家人才管理改革试验区建设。深化科技体制改革，建立多元化科技投入机制，完善创新人才和科研成果评价、激励机制，全社会研发投入占GDP比重达到4%。到2020年，基本建成国际化创新型城市。

　　二是全面深化改革开放。高标准建设横琴自贸试验区，对接国际高标准投资贸易规则，深化和完善准入前国民待遇加负面清单管理制度。分类推进国有企业改革，规范有序发展混合所有制经济。完善服务民营经济发展的平台和体制机制，消除各种隐性壁垒。推进简政放权，进一步精简审批事项和审批环节，健全审批后监管制度。全面建设法治政府，用好地方立法权，完善两法衔接。构建全民共建共享的社会治理格局，基本建成社会建设示范市。同时，把握国家“一带一路”战略实施机遇，推进珠港澳深度合作，打造CEPA升级版，与澳门联手建设中国和拉美国家经贸合作平台，构建开放型经济新格局。到2020年，基本形成市场化国际化法治化发展环境。

　　三是加快建设“三高一特”现代产业体系。对接中国制造2025，实施智能化技术改造，建设格力国际智能制造基地和高新区机器人产业园，打造省智能制造示范基地。依托西部先进制造基地，聚焦海洋工程、生物医药、航空航天和绿色智能交通等重点产业，形成1-2个千亿级先进制造产业集群。依托国家自主创新示范区，建设国家高技术产业基地和省战略性新兴产业基地，申报保税区国家级增材制造技术创新中心。依托十字门中央商务区等重点区域，加快发展金融、物流、会展、跨境电商、文化创意等高端服务业，建成对接港澳、辐射珠江西岸的科技创新中心、区域物流中心和总部经济基地。依托海域海岛资源，打造粤港澳游艇消费基地和一批国家级休闲渔业示范基地。到2020年，基本建成以智能制造为龙头的“三高一特”现代产业体系。

　　四是推进国际宜居城市建设。优化城市发展空间格局，推进“五规融合”，着力打造港珠澳国际都会区。创新生态文明建设体制机制，成功创建全国首批生态文明示范市、全国水生态文明城市和国家森林城市，基本形成低碳生态发展模式。加大工业源、尾气排放和扬尘等治理力度，确保空气质量始终保持在全国前列。改进自来水处理工艺，改造老旧供水管网，提升城市供水品质。论证建设直饮水工程的可行性。完成前山河等流域整治与生态修复工程，初步建成淇澳红树林湿地公园、凤凰山森林公园和黄杨山森林公园，建设一批城市林荫道、滨海景观道、绿色生态水道、环山健康步道和乡村风情道，形成较为完善的慢行系统。建设中欧低碳生态综合试点城市，打造前山河绿色建筑、南湾绿色交通等一批示范项目，公交车100%使用清洁能源，推进高新区国家级生态工业示范园区和高栏港循环经济试点园区建设。实施“互联网+”行动计划和大数据发展战略，“政府WIFI通”覆盖公共服务场所，推进智慧交通、智慧城管、智慧政务、智慧社会服务等项目建设，成为珠三角智慧城市群的重要一极。到2020年，基本接近国际宜居城市。

　　五是推进交通枢纽城市建设。推动港珠澳大桥建成通车，主动联接深中通道，谋划建设深珠城轨，主动融入珠江口国际湾区。加快推进广佛江珠城际轨道建设，建成金琴快线、兴业快速路等内通外联工程，实现15分钟内主要交通节点上高快速路。建成金海大桥、香海大桥、洪鹤大桥及其连接线，力争建成市区至机场城际轨道。加大公交投入，增加运营线路，建成一批综合性公交枢纽站场，公交机动化分担率超过45%，中心城区交通拥堵问题得到明显改善。建设第三代、第四代国际港口，建成高栏港疏港铁路专用线二期，打造通江达海的珠海港港口枢纽。推动开放珠海机场国际口岸，启动第二跑道建设，打造复合型国际干线机场和货运枢纽机场。到2020年，建成以港珠澳大桥为龙头的立体综合交通枢纽城市。

　　六是构建均衡的发展格局。推进西部生态新区建设，基本形成西部生态新城核心区的框架，完成金湾、斗门和平沙新城起步区的建设。建设更高水平的幸福村居，培育一批水产品养殖业、生态休闲旅游农业和电商农业名优品牌，公交车覆盖全部行政村。实施“东优西拓”的人口发展策略，基本建立以合法稳定住所和职业为户口迁入基本条件、城乡统一的新型户籍管理制度。提升珠江口西岸核心城市的功能和辐射带动作用，在交通、水资源、产业、环保、旅游和民生等领域开展珠中江阳紧密合作。到2020年，基本实现东西区协调发展格局。

　　七是建设和谐幸福珠海。以西部地区为重点，提高基本公共服务整体水平和均衡化水平。实施更加积极的就业政策，城镇登记失业率控制在3.2%以内，实现居民收入增长和经济发展同步。创建教育现代化示范市，进一步缩小区域、城乡、校际间差距，等级幼儿园达到90%以上，规范化普惠性幼儿园达到80%以上。健全基本养老保险制度，建立城乡一体的医疗保险体系，建设覆盖城乡的多层次养老服务体系。提高城乡居民最低生活保障、农村五保供养等底线民生保障水平。健全面向低收入家庭的住房保障制度，扩大住房保障覆盖面。新增医疗卫生资源向西部、海岛和基层倾斜。深化公立医院改革，实行分级诊疗，鼓励发展非营利性民营医院和高端民营医疗机构。全面推进市、区、镇（街）、村（居）四级公共文化体育设施建设，基本建成覆盖城市社区的市民艺术中心。构建社会安全、生产安全、食品药品安全和环境安全的城市管理体系。到2020年，率先实现基本公共服务均等化，教育、文化、医疗等达到较发达水平。

　　三、2016年工作安排

　　2016年是“十三五”的开局之年，也是珠海实施创新驱动发展战略、加快转型发展的关键之年。综合研判，经济长期上扬的态势和短期下行的压力并存。从大环境看，世界经济复苏乏力，国内经济下行压力持续加大，发展面临的外部环境复杂严峻。从珠海自身情况看，固定资产投资连续几年高位运行后增速放缓，消费新热点不多，外贸受制于国际市场需求疲弱，实体经济新增投资动力不足，稳增长面临的压力和挑战非常大。但同时更要看到，随着全局性改革开放的深化和珠海特有发展要素的有效叠加，我们承接国内外发展资源的机遇前所未有。特别是，随着供给侧结构性改革在全国的铺开，我们的发展优势将更加突显。因此，我们既要增强忧患意识，更要坚定战略自信，把工作做得更扎实一些，努力完成全年目标任务，确保“十三五”开好局、起好步。

　　2016年政府工作的总体要求是：全面贯彻党的十八届三中、四中、五中全会和中央经济工作会议、城市工作会议精神，深入系统学习贯彻习近平总书记系列重要讲话精神，认真落实省委十一届五次、六次全会和市委七届六次、七次全会部署，牢牢把握创新、协调、绿色、开放、共享的发展理念，主动适应新常态，深入实施创新驱动发展战略，扎实推进供给侧结构性改革，努力构建市场化国际化法治化的发展环境，加快交通基础设施建设，统筹推进横琴自贸试验区、自主创新示范区和西部生态新区建设，努力建设国际宜居城市，加快推进基本公共服务均等化，促进经济稳定增长，保持社会和谐稳定。

　　建议2016年经济社会发展的预期目标和约束性指标是：地区生产总值增长8.5%-9%；规模以上工业增加值增长9.5%；社会消费品零售总额增长11%；固定资产投资增长15%；外贸进出口持平，其中出口增长1%；一般公共预算收入增长8%；全体居民人均可支配收入增长8%；居民消费价格涨幅、城镇登记失业率分别控制在3%和3.2%以内；节能减排降碳约束性指标完成省下达目标。

　　需要说明的是，与往年相比，我们适当调低了2016年的经济增长预期，主要是充分考虑了2016年经济整体下行的压力，同时也为供给侧结构性改革留出了空间。从全国全省的情况看，在新常态下确立这样的增长预期目标，是积极的，也是切合珠海实际的。GDP增长提出了8.5%-9%的区间目标，是综合考虑了各种不确定的因素，有利于保持经济增长的弹性。

　　围绕实现2016年的预期目标，建议重点安排八项工作：

　　（一）促进经济稳定增长，实现“十三五”良好开局

　　扩大基础设施、产业、民生等重点领域的有效投资。安排重点项目337项，年度计划投资494.2亿元。建立制度化的领导干部分类协调、督导重点项目机制，加快推进108项续建项目，力促53个在建项目建成投产，新开工91个项目。建立未来三年的政府投资项目库，提前开展前期工作。开展更有针对性的招商引资，办好第十一届中国国际航空航天博览会、2016中国（珠海）国际海洋高新科技展览会、珠海国际游艇展和珠海国际智能电网展等展会。

　　培育新消费热点。出台扶持新业态发展的政策。修订促进电子商务发展的措施，建设跨境电商公共平台，推动横琴自贸试验区和保税区开展跨境电子商务进出口业务，推进与阿里巴巴、聚美优品、运易通等电商的合作项目，建成一批农村淘宝村级服务站。推进港珠澳世界级旅游休闲目的地建设，密切珠港澳旅游合作，创新旅游联合推广机制，办好珠中江、珠中澳、广深珠等旅游推介会。加快长隆第二主题公园、海泉湾二期等在建项目建设，推动航空大世界等新签约项目动工，开展境外旅客购物离境退税政策试点，争取国家支持建设“横琴国际休闲旅游岛”，规划建设一批游客集散中心和咨询服务中心等公共服务配套设施。加快商务信用建设，大力打击假冒伪劣违法行为。

　　促进外贸稳定增长。修订外贸稳增长调结构扶持政策，启动加工贸易转型升级三年行动计划，组织和发动企业参加国内外重大展会。拓展内外贸航线和多式联运业务，推动珠海港与巴西维多利亚港开通直航航线，继续申报高栏港综合保税区，建设拉美综合保税枢纽仓储物流中心。继续推进跨境工业区转型。发展外贸综合服务、旅游购物出口等新业态。建设服务贸易特色出口基地。建成电子口岸和国际贸易“单一窗口”。

　　扶持实体经济发展。出台支持现有企业发展的政策。“一企一策”培育大型骨干企业。开展小微企业“幼狮计划”，落实“小升规、个转企”扶持政策，新增43家规模以上工业企业。深入调研影响民营经济发展的问题，加大对民营经济的扶持力度，推动民营中小企业公共服务平台上线运行。

　　（二）深入实施创新驱动发展战略，建设珠江西岸创新中心

　　完善创新生态环境。修订出台《珠海经济特区科技创新促进条例》。编制自主创新示范区建设实施方案、发展规划纲要、空间调整规划和政策实施意见。完善科技创新的体制机制，建立市统筹扶持公共平台、区扶持企业和项目的工作机制。引进股权投资基金和创业投资基金。建立科研诚信“黑名单”。成立民商事调解中心和高新知识产权仲裁中心。推进标准化和质量品牌建设，完善质量发展规划，建成全国质量强市示范市。

　　促进科技金融融合发展。建设众创金融街。依托横琴国际知识产权交易中心，开展知识产权交易和知识产权融资业务。推动珠海产权交易中心开办非上市企业股权托管、融资业务。加快“新三板”华南服务基地建设。启动科技创新券试点。探索创新产品与服务远期约定购买制度，推动科创投公司正式运营。

　　全面落实高层次人才倍增计划。优化人才政策体系。争取国家、省在珠海率先开展技术移民制度试点。加快横琴人才管理改革试验区建设，推进横琴与港澳执业资格互认，实现港澳专业人士在横琴便利执业。

　　加强创新载体建设。实施高企培育专项行动，设立高企专项扶持资金，力争高企数量超过500家。推进清华珠海创新中心等新型研发机构建设。开工建设横琴国际科技创新基地。编制孵化器发展规划，建成珠海信息港等项目，新增3家市级创业孵化基地，资助一批优秀创业项目。支持驻珠高校和企业建设创客空间、孵化基地和劳模职工创新工作室。与中山大学、吉林大学共建理工类学院、学科和研究生院，支持暨南大学等院校面向战略性新兴产业提升和新建理工类学院。推进省科学院海洋工程装备、航空航天和生物医药技术等三所研究院建设。依托公共技术服务平台，推动创新成果的标准化转换和应用。

　　（三）以横琴自贸试验区为龙头，全面深化改革开放

　　系统推进横琴自贸试验区制度创新。对标国际投资贸易规则，落实市场化国际化法治化营商环境建设的32项工作。优化“三个零”政府服务机制，实现政府服务电子化、清单化、标准化。加快建设横琴“廉洁岛”。开展口岸查验机制创新试点，争取在监管互认、信息共享、执法互助等方面取得实质突破。实施澳门车辆在横琴与澳门间便利进出政策。推进金融开放创新，落实人民银行关于金融支持自由贸易试验区建设的指导意见，扩大人民币跨境使用，深化外汇管理改革，深化以粤港澳为重点的区域金融合作，提升金融服务水平，强化风险监测与管理。推动消费金融公司、与港澳合资的全牌照证券公司等法人金融企业落地。延伸建设十字门中央商务区板块，发挥自贸区的辐射带动作用，推动横琴与各区联动发展。

　　深入推进经济体制改革。促进商事登记便利化，建成“一照一码”登记全程电子化服务系统，推行商事主体登记注册“同城通办”，完善行政许可协同审批和后续监管协同执法改革。深化投融资体制改革，修订政府投资项目管理条例，推广公共服务领域政府和社会资本合作模式，开展企业投资项目直接落地改革试点。深化国有企业改革，培育改组国有资本投资运营公司，加大企业重组力度，引进战略投资，推进资产证券化和企业市场化机制的建设。完善零基预算，扩大部门预算现场联审试点，细化部门预决算公开。编制中期财政预算规划。

　　提升行政服务效率。下放第二批市级行政管理事权，科学调整市、区两级编制结构。出台新的政府职能转移目录，取消一批职业资格行政审批事项，清理规范行政审批中介服务，制定市场主体许可经营项目及经营场所监管清单。编制行政审批事项办事指南，启动“一门式”政务服务改革试点。建立完善市、区两级政务信息资源共享平台，实现四级电子政务网络光纤全覆盖。

　　完善以民为本的社会治理体制。建设一批镇（街）、社区社会服务中心，对接政府购买服务和社会工作专业服务，探索群众“点单”、社会力量“接单”、政府“买单”的服务模式。开展民警分类管理改革，健全警务辅助人员管理制度，建立以平安指数为核心的社会治安综合治理考评体系和治安情况通报机制，构建扁平化、实战化现代警务运行机制，提高路面见警率。深入推进户籍制度改革，调整户口迁移政策。高度重视反恐防暴工作，加快公共危机预防和应急处置体系建设，编制公共安全与危机应对“十三五”规划。完善安全生产责任体系，推进高栏港区安全发展示范园区建设，加强大型危险化学品管道和储存器的风险管控。

　　积极参与“一带一路”建设。打造中国和拉美国家经贸合作平台。创建中拉电子商务产业基地。推动川贵粤-南亚物流大通道等项目建设。建立更务实的珠港澳合作工作机制和定期会晤磋商机制，推进粤港澳游艇“便利行”。积极推进珠中江阳一体化重点项目。

　　（四）扎实推进供给侧结构性改革，增强经济持续增长动力

　　淘汰落后产能。制定重污染高耗能企业搬迁三年行动计划，推动红塔仁恒等企业搬迁改造。继续推进“三清”工作。支持企业兼并重组。落实工业企业技术改造三年行动计划，支持企业扩产增效、智能化改造、设备更新和公共服务平台建设，全年完成技术改造投资100亿元以上。建设格力国际智能制造基地和高新区机器人产业园，实施“机器人应用”计划。开展两化融合管理体系贯标试点，支持企业申报工信部互联网和工业融合创新试点。

　　促进房地产市场平稳健康发展。动态监控评估房地产市场情况。放宽住房公积金贷款条件，开展公转商贴息贷款业务，推进公积金异地互认使用。适度增加中心城区土地供应，推动房地产开发重心向新城新区转移。建立房地产开发企业和物业服务企业信用管理系统。新开工2300套保障性住房，推出2500套公共租赁住房。

　　补齐农业供给短板。发展高品质、高效益、高附加值的特色农业。充分发挥农控集团、台创园作用，提升农业的品种、品质和品牌。建成农产品电子商务展销平台，启动农产品质量安全监测及物流中转中心、水产城等项目建设。规划建设广东（珠海）现代种业发展中心，开工建设展览检测综合试验楼等项目，办好中国（珠海）台创园农业博览会暨农业项目对接会。发展农村金融，推进农户信用信息采集及信用村、信用户评定工作。开展涉农保险创新，推进政策性水产价格指数和风灾指数保险试点工作。

　　“营改增”全面扩围，扩大小型微利企业减半征收企业所得税范围。继续清理一批行政事业性收费。防范化解金融风险，以加强新兴金融业态监管和防范打击非法集资为重点，出台监管措施，健全工作机制，推动金融行业加强自治自律。

　　（五）打好交通攻坚战，综合治理核心城区交通拥堵

　　完善高快速路网和市政路网。全面开工建设香海高速、洪鹤大桥，新开工建设坦洲至香海高速连接线、金海公路大桥、鹤洲至高栏港高速，建成洪湾互通二期主线工程。完成兴业路北延线、港湾大道快速化改造前期工作，建成月环支线南延段，全面开工建设金琴快线。启动园山路、翠海路等升级改造，打通兰埔路、新海燕桥、造贝路等一批断头路。

　　实施中心城区交通改善三年行动计划。综合运用完善路网、道路挖潜、绿色出行、管理引导四大策略和一系列具体措施，缓解主城区交通拥堵。完善道路停车咪表的规划建设，优化咪表功能设置。完成港珠澳大桥通车后交通组织、通行政策和通关模式的研究。围绕深中通道建设，衔接珠中高快速路网和市政路网。研究小汽车区域限行政策。

　　加大公共交通发展力度。加快珠机城轨一期建设，开工建设二期工程。争取推动广佛江珠城际轨道年内开工，调整完善有轨电车线路规划，论证实施二期建设工程。增加公交投放，优化公交线网，推行社区、村镇、工业园区、大学园区的小巴服务。推进部分道路公交专用道和一批枢纽站场建设。实施公共交通换乘优惠。改造56公里自行车道，建成二期公共自行车租赁系统。

　　加强港口、机场、口岸建设。推进黄茅海航道、疏港铁路专用线二期建设。开工建设15万吨级粮食码头和石油码头。推进珠海机场航站楼升级改造，建成通用机场。加快港珠澳大桥珠海口岸、珠港澳物流合作园首期通关中心建设。开通万山港口岸香港航线。

　　（六）提升城市功能品质，夯实国际宜居城市基础

　　构建可持续发展的城市空间格局。完善“五规融合”一张图表编制，建立项目协同审批机制。推进15个海绵城市建设工程项目，新开工建设25公里综合管廊。推进“一河一带两轴两镇三港三心”更新改造，集中推进香洲城区的旧村庄和旧厂区的连片改造，完成主城区100万平方米老旧小区改造任务，基本完成洪湾渔港建设，建成上冲TOD小镇，开工建设拱北口岸商贸中心南区、九洲港货运码头和香洲港等改造项目。开展“建筑质量和安全年”活动，全面执行绿色建筑标准。推进“四极两翼”蝶形电网建设。

　　巩固和提升珠海的生态优势，加快创建全国首批生态文明示范市。推进中欧低碳生态综合试点城市建设。修编生态文明建设规划。实行最严格的水资源管理制度，开展61条河涌清淤截污。建成前山河流域旧村截污、第一批管网和前山污水厂一期工程，启动第二批管网和大型拦污栅工程。深入实施大气污染防治行动计划，继续淘汰黄标车，开展工地和道路扬尘专项治理。抓好横琴东岸、口岸人工岛、野狸岛生态岸线修复和重点区域山体修复工作。出台生态控制线管理规定，开展生态控制线划桩定界工作。建立海洋生态补偿机制，推动海洋生态保护和环境治理。设立生态文明指数珠海发布中心。制定排污权有偿使用和交易管理办法。建立企业环境信用公开机制。

　　启动“公园之城”建设。开工建设3个森林公园、2个湿地公园，建成33个乡村绿化美化点，新建香山湖公园、改造提升野狸岛公园、海天公园等都市特色公园，构建四大公园体系。推进城市绿廊和林业生态工程建设，绿化美化一批桥梁和隧道，新建70公里绿道、8660亩碳汇林和16.8公里生态景观林。创建国家森林城市。

　　提升市容环境卫生水平。启动城乡生活垃圾分类和餐厨垃圾收运处理一体化项目。建成中信生态园首期环保生物质热电工程，启动二期工程。启动东部污泥处置中心搬迁工程，开工建设西部污泥处置中心。编制公共厕所专项规划，新建和改造50座旅游公厕。开展违法建筑专项整治。完成城镇水浸黑点改造。提高环卫工人工资待遇。

　　整体推进智慧城市建设。编制智慧珠海2016-2018三年行动计划，统筹建设医疗卫生、教育、交通、文化旅游等民生领域的智慧应用项目。建立公共服务平台建设和应用的统筹协调机制。建成智慧珠海云计算中心、新一代公安数据中心、智慧交通信号协调控制系统等项目，整合智慧交通、治安监控和数字城管系统，构建大数字城管平台。升级市民和企业专属网页，实现市民和企业通过一个平台网上办理各类事项。

　　（七）加快西部地区开发建设，促进区域协调发展

　　加快先进装备制造产业带建设。建设海洋工程装备国家新型工业化产业示范基地，争取创建国家级增材制造技术创新中心。促成中海油管道制造、太平洋粤新海洋工程船等项目竣工投产，华彬通用航空基地等项目落地建设。

　　全面铺开西部生态新城起步区建设。理顺西部生态新城建设管理体制。全面开工建设主干路网，启动部分次干道建设。完成富山新城马山园区、三村园区等5个园区服务中心建设，启动西部中心片区和金湾航空新城起步区的公共服务设施建设。

　　完善西部地区基础设施。升级改造42公里县道，开工建设S272莲洲至井岸段、珠峰大道等改造工程，完成井岸大桥、南水大桥等桥梁加固，启动上横大桥、尖峰大桥加固工程。完成乾务赤坎大联围、小林联围海堤加固达标等水利工程建设任务，开工建设金湾大门口水道引水工程，加快推进白藤大闸重建等8个水利项目前期工作。

　　推进幸福村居创建工作。继续实施村居“四整治一美化”工程，农村生活污水处理覆盖所有自然村，农村生活垃圾实现100%无公害处理。推进农村土地承包经营权和农村集体建设用地、留用地使用权确权登记、农村集体经济社区型股份合作制等改革，完善以自然村为单位的村民自治机制。分批解决农村集体留用地落地问题。打造斗门、金湾两个省级农村示范片区，率先完成一批市级新农村建设示范片区。

　　在加快西部地区发展的同时，大力促进海岛地区开发建设。整体谋划开发海岛海域旅游，完善开发规划，全面摸查海岛生态要素。完善海岛供水、供电等基础配套，推进东澳水库二期扩容、桂山海上风电场、万山海岛新能源微电网示范项目。建设东澳岛客货运码头、唐家港陆岛交通补给码头。探索庙湾岛等无居民海岛旅游开发模式。办好万山群岛海鲜美食节、海岛妈祖旅游文化节等活动。建设海洋现代养殖基地，发展深海养殖。探索建设中国-东盟海产品交易中心。增加市区至海岛的公交班轮密度。

　　打好市内扶贫攻坚战。开展低收入群体和困难家庭基本情况摸底调查，制定具体帮扶方案。出台医疗救助、就业扶持、教育资助等针对性的帮扶政策和标准。加快改善西部地区及海岛困难群体居住条件。开展扶贫志愿者行动计划，搭建社会扶贫力量与困难群体的对接平台。同时，做好对口帮扶凉山彝族地区、对口支援甘孜藏区、巫山三峡库区、西藏林芝贫困县和精准扶贫阳江、茂名工作，完成对口帮扶阳江三年目标任务。

　　（八）推进基本公共服务均等化，补齐民生事业短板

　　提升基础教育整体水平和均衡化水平。设立促进西部地区教育均等化专项资金。理顺高中教育管理体制，全市公办普通高中由市教育局统一管理。推进集团化办学，组建幼儿园一体化发展联盟、义务教育学校联盟和普通高中学校联盟。完成香洲区对海岛学校的整体托管。出台促进融合教育发展实施办法。全面实施中小学校长职级制，启动教师编制和岗位聘用改革，探索建立校长、教师退出机制。选拔一批优秀学校管理干部和教师到农村中小学校任职、支教。推进技工学校、省实验中学珠海金湾学校、白藤东小学等一批教育基础设施建设，建成金湾航空城小学、香洲第十三中学、第二十三小学等学校。

　　提高医疗卫生服务水平。全面启动卫生强市和健康珠海建设。推动公立医院去行政化，建立法人治理结构，改革人事薪酬制度。设立西部地区及海岛医疗卫生发展专项资金，实行镇卫生院和农村卫生服务中心机构建制一体化，把万山海岛医疗卫生机构成建制整体划入市人民医院。鼓励公立医院医师参与社会办医，建立民营社区卫生服务机构补偿和退出机制。推进基层医疗机构中医药服务标准化建设，开展高血压、糖尿病分级诊疗，实现家庭医生团队全覆盖。出台《引进高层次卫生专业人才管理办法》。完成健康城市基线调查，建设20个达标的健康细胞单元。建立健全重大传染病联防联控机制。加快建设新市妇幼保健院、市慢性病防治中心、市第二中医院（侨立中医院）扩建和平沙医院改扩建等项目，推动省人民医院金湾医院投入运行。实施“全面两孩”政策，加快公立医院妇科、儿科建设。推进控烟立法。

　　打造“十分钟文体圈”。构建面向社区的基本公共文化服务体系，建设覆盖社区的市民艺术中心。建设珠海市图书馆总分馆。完成数字农家书屋建设任务。分批向社区开放公办学校体育设施。建成珠海大剧院、博物馆和规划展览馆。制定政策，将符合条件的民办文化场馆纳入公共文化服务体系。申办全国帆船锦标赛，办好珠海WTA超级精英赛等城市品牌活动。开展足球、网球进校园活动。制定社会科学普及规划。加强公民道德和精神文明建设，深入实施巩固提升文明城市三年行动计划。出台促进文化创意产业发展的政策。

　　完善以养老保障为重点的社会保障体系。大力发展社会养老，出台养老设施布局规划和养老服务业发展方案，制定养老服务标准和补贴政策，推动智慧养老服务，继续推动老年大学进社区，探索医养结合养老模式，建设以居家为基础、社区为依托、机构为补充的养老服务体系。把居民养老保险基础养老金标准提高到每人每月360元，低保标准提高到每人每月620元，公共卫生服务经费财政补助标准提高到每人每年50元，基本农田保护补贴标准提高到每亩150元。启动青年大学毕业生住房保障计划。组织大学生就业创业培训1万人次，完成5万人次全民技能提升培训，城镇新增就业4.5万人。制定残疾人“十三五”规划。

　　全力解决市民关注的热点问题。加强城市供水取水水源的保护和库区的水质管理。开展常规食品检测，为市民提供便捷的食品安全检测服务。新增30所校园食品安全科技工程试点学校。建设城市慢行步道系统，启动环山步道、滨海景观步道、城市林荫道建设。加快燃气管道建设，逐步降低用气价格。

　　办好十件民生实事。一是实现城乡居民与企业职工医疗保险门诊、住院报销同等待遇，将城乡居民医疗保险财政补贴标准由每人每年400元提高到480元，住院报销比例提高到90%，报销限额由每年40万元提高到62万元。二是推进民生水利工程实施，完成9.5公里斗门区鹤洲北海堤达标加固和36公里白蕉联围堤顶路面加固建设。三是提升社区卫生服务中心的医疗服务能力，每个农村卫生服务中心配备1至2名全科医生，运行经费由每年10万元提高到30万元，实现小病不出社区。四是完善人行过街等配套设施，动工建设S272湖心路段2座人行天桥、珠海大道西段3座人行天桥和情侣路南段（昌盛路至横琴大桥段）12座人行地道。五是建成东西部20.9公里公交快速化工程（BRT）及前山、湖心路公交换乘枢纽。六是完善基层公共服务综合平台建设，在全市318个村（居）公共服务站全部设立综合服务窗口，开展代办服务，实行一站式办理，面向基层的公共服务事项全部进驻网上办事大厅。七是在车站、机场、码头、医院、行政办事大厅、公共文化体育场馆、公园等公共场所为市民提供全覆盖的免费WIFI接入服务。八是提升社区文化体育服务水平，完成15个市民艺术中心、110个村居文化中心和34个社区体育公园建设。九是实现全市月保教费不超过1000元的公办及民办普惠性等级幼儿园比例达到50%以上。十是改造提升西部地区农村道路，实现西部地区50人以上自然村村村通水泥路，完成86公里的机耕路建设。

　　要完成年度工作任务，必须进一步加强政府自身建设，提高政府的执行力和公信力。我们将大力推进法治政府建设，加强和改进政府立法，建立行政决策专家咨询库，推进综合执法体制改革，健全政府绩效考评机制，将政府工作全面纳入法治轨道。我们将深化“三严三实”专题教育，巩固党的群众路线教育实践活动成果，严格落实“八项规定”，坚持“三个区分”原则，努力营造敢于担当、勇于作为的干事氛围。我们将严格落实党风廉政建设“两个责任”，加强廉政风险防控和审计监督，让群众切实感受到政府作风的变化。我们将更加注重政府的公共关系，构建政府与市民更加便捷的沟通渠道，办好政府门户网站和官方微博、微信，健全常态性的新闻发言人制度，促进信息公开对称，更好地吸纳社会和民间智慧。我们将认真落实市人代会及其常委会的各项决定，定期向市人大常委会报告工作，自觉接受市政协的民主监督，建立政府约见人大代表、政协委员，以及部门对口联系市各民主党派和无党派人士的工作机制，办好人大议案、代表建议和政协提案。

　　各位代表，珠海正迎来新一轮发展的黄金时期，前景催人奋进。让我们在市委的坚强领导下，坚定信心，迎难而上，扎实工作，圆满完成2016年各项目标任务，共同创造珠海更加美好的明天！
